# The TEMPO program and the future of digital mental health regulation in the US

**DOI:** 10.1038/s41746-026-03464-x

**Published:** 2026-11-03

**Authors:** Acacia C. Parks, Aubrey Shick

**Affiliations:** 1 Avania, Marlborough, MA USA; 2 Launch and Logic, Boulder, CO USA

**Keywords:** Business and industry, Engineering, Health care, Scientific community, Social sciences

## Abstract

The high burden of traditional FDA clearance drives many novel, AI-enabled mental health devices into unregulated consumer markets. The new FDA-CMS TEMPO pilot program establishes a vital regulatory middle ground, allowing companies to commercialize using a Quality Management System without immediate premarket authorization. This commentary explores how TEMPO serves as a sandbox for real-world data, offering a blueprint to reshape the future of US digital health regulation.

It is an exciting time for digital mental health, yet FDA premarket clearance for Software as a Medical Device (SaMD) remains notoriously unyielding. To date, digital therapeutics (DTx) devices such as Rejoyn, Daylight, and MamaLift have cleared a high evidence bar with randomized, sham-controlled clinical trials as evidence of safety and effectiveness. Furthermore, existence as a medical device requires developing software and operating under a Quality Management System Regulation (QMSR) compliant Quality Management System (QMS). Over 10 years of interactions with review teams within FDA’s OHT 5 (Office of Neurological and Physical Medical Devices – which is responsible for mental health indications) confirm that these foundational expectations have scarcely changed. Companies with well-understood technologies will continue to tread that somewhat well-trodden path (paved by Pear, Click, Big Health, Curio, and others) and are making headway earning marketing authorization for their DTx devices. While this commentary is not about them, a baseline understanding of the traditional gauntlet is essential to appreciate why the FDA’s newly launched Technology-Enabled Meaningful Patient Outcomes (TEMPO) for Digital Health Devices Pilot program marks such an interesting and relatively radical development in the US regulatory landscape for digital mental health.

The focus of this commentary is on the companies making DTx devices that leverage less-understood technologies—especially those that generate non-deterministic output (such as LLM-based chatbots). The barrier to entry for FDA clearance is particularly high for companies developing generative AI-enabled products. Beyond the requirements outlined above, these sponsors face the additional challenge of establishing evidence and performance expectations in areas where regulatory standards are still emerging. FDA has indicated that generative AI introduces new risks that likely require additional oversight, mitigations, and controls^1^. However, the agency has not yet established a comprehensive framework defining how those risks should be evaluated across applications. Instead, FDA has signaled an intention to apply an evolving, risk-based approach, with policy and regulatory expectations continuing to develop through individual product reviews. The path is highly indeterminate, and the only path forward is through multiple FDA interactions, each of which is a multi-month process. Taken together, these challenges could mean that establishing a traditional regulatory pathway to market for LLM-based medical devices may take years.

For these reasons, many novel mental health devices do not pursue FDA authorization, but instead exist in consumer or B2B-facing solutions that do not overtly advertise themselves as treatments, even though in many cases, under close analysis, they are performing many activities that a therapist or other care provider might. These companies have been able to avoid FDA medical device regulation by relying on exclusions established by Section 3060 of the 21st Century Cures Act, which created FD&C Act §520(o), as well as FDA’s “enforcement discretion” policies for certain lower-risk products^2^. These policies are reflected in FDA’s Device Software Functions and Mobile Medical Applications (DSF-MMA) guidance^3^, and have allowed products making relatively limited claims (e.g., helping users “maintain their behavioral coping skills”) to reach the market without premarket review. Companies in the US can therefore make limited device claims without triggering FDA enforcement because FDA has essentially declared that they do not intend to enforce there. Specifically, the DSF-MMA guidance^3^ states, “Consistent with FDA’s existing oversight approach that considers functionality of the software rather than platform, FDA intends to apply its regulatory oversight to only those software functions that are medical devices and *whose functionality could pose a risk to a patient’s safety if the device were to not function as intended*.**”** For software functions that do not pose such a risk (and many such examples are listed in the guidance), “FDA does not intend to enforce requirements under the FD&C act” (which includes reporting, QMS, GMP, and so on). Because it is possible to commercialize in a limited way without FDA clearance, and because the barrier to entry for FDA clearance is so high, many companies with novel technologies have chosen to sit outside regulation indefinitely. And because they are operating out of FDA oversight, they are not held to regulatory standards when it comes to safety, effectiveness, quality or reporting. The existence of mental health interventions, marketed in a completely unregulated environment with implied treatment capabilities, is the source of great concern among clinicians and researchers alike^4,5^—but the reasons companies are choosing to go this route is no great mystery. There are, at present, only two options: high evidence, high validity, high burden, or low evidence, low burden, validity unknown. There is no right-sized, middle path.

The TEMPO program presents an important middle ground. TEMPO is an FDA-CMS collaborative effort, offered in the broader context of the Advancing Chronic Care with Effective, Scalable Solutions (ACCESS) model^6,7^, which introduces outcome-aligned payments for technology-supported care. Importantly for the current paper, in a landscape where there has thus far been no commercial advantage to obtaining FDA authorization, the TEMPO pilot is a figurative oasis in the desert. The TEMPO program^8,9^ allows selected companies to go to market in Medicare without a 510(k) or De Novo authorization under enforcement discretion (but notably, a different usage of the enforcement discretion mechanism than we have seen described in DSF-MMA; see below). Sponsors then generate Real-World Data (RWD), which can be leveraged to incrementally secure formal clearance. While it is unclear what role RWD will play in the overall evidence package for clearance (i.e. will sponsors still need a sham controlled study?), the concept that RWD will even factor into an initial clearance is already without precedent in the world of DTx.

Companies leveraging large language models to deliver personalized mental health services through conversational interfaces are, in our view, the natural audience for TEMPO. Their unprecedented scalability and potential to improve access to care are well-matched by novel questions regarding safety, effectiveness, and real-world performance, making TEMPO a particularly suitable regulatory home for development and evaluation. While FDA has indicated that it will select up to 10 participants for the mental health track of TEMPO, practical experience working with companies interested in TEMPO participation reveals a much tighter bottleneck, which begs the question: what types of companies is FDA looking for? FDA has been clear that generative AI raises unique questions regarding safety, validation, and ongoing oversight. For that reason, TEMPO’s emphasis on reasonable assurance of safety might seem, on its face, to disadvantage LLM-enabled products. Our experience has been the opposite. The only applications we are aware of receiving active FDA engagement have been those leveraging large language models. This signals, at least informally, that the Agency is deliberately trying to bring innovative technologies with unclear regulatory pathways into the fold.

It is important to note that the operationalization of “enforcement discretion” in TEMPO diverges from that of the DSF-MMA policy in several key ways, and it is in that distinction that the TEMPO program becomes truly exciting for digital mental health. Under DSF-MMA, which is a relatively broad-swath enforcement discretion approach, a Quality Management System (QMS) is not legally required, though the Center for Devices and Radiological Health (CDRH) unofficially encourages companies adopting them as best practice (personal communication, multiple sources). In contrast, while TEMPO also invokes the concept of enforcement discretion to allow companies to go to market with their devices without premarket authorization, FDA has clarified that it expects companies in TEMPO to have a working, QMSR-compliant QMS. This is a fundamental departure from the enforcement discretion of DSF-MMA.

The use of an enforcement discretion that does not exclude QMSR compliance in TEMPO suggests that FDA is exploring middle paths between the enforcement discretion “wild wild west” of DSF-MMA and a traditional 510(k) or De Novo process. One interesting regulatory parallel is the 510(k) exemption framework^10^, under which manufacturers may bring certain products to market without premarket review provided their claims remain within the scope of an established product code. This flexibility does not eliminate regulatory oversight; manufacturers remain subject to General Controls, including FDA registration and listing, a functional QMS compliant with QMSR, and FDA inspection. In some ways, 510(k) exemption is similar to DSF-MMA enforcement discretion; companies self-register and are able to commercialize freely with some constraints to marketing claims. In other ways, it is more rigorous; sponsors must have a QMS, and could at some point be inspected for compliance with the marketing claims guidelines of the product code (unlike DSF-MMA enforcement discretion, where FDA specifically “does not intend to enforce requirements under the FD&C Act”). The bar of accountability is higher, but still a substantially lower bar than a premarket authorization. Notably, there are no 510(k) exemption product codes that allow LLM-based devices to self-register, so in this scenario, it is most likely that 510(k) exemption will replace the traditional 510(k) as the most common pathway to regulatory oversight for risk-managed non-gen-AI based mental health devices, while programs like TEMPO will continue to evolve to support the more innovative products that require premarket authorization.

What, then, might the future of digital mental health regulation hold? We would argue that FDA can use the TEMPO pilot as a controlled sandbox to test out the real-world safety mechanics commercializing mental health DTx with a QMS but without premarket authorization, creating the blueprint to roll out a formal 510(k) exemption framework for more defined, traditional DTx tech down the road. If TEMPO goes well, devices using well-understood technology could self-register as 510(k) exempt, while devices using innovative technology will continue to rely on programs like TEMPO to define and accelerate regulatory progress. This is consistent with how relevant existing 510(k) exemption codes are configured—21 CFR 882.1470^11^ “Computerized Cognitive Assessment Aid,” for example, has a 510(k) exempt product code (PTY), but explicitly calls out that certain device features (e.g. analysis of continuous signals like auditory inputs – which typically requires AI) excludes a device from 510(k) exemption. Notably, the existing DMHT codes are tied to 510(k) clearance off of 21 CFR 882.5801^12^, and 510(k) exempt products would only be eligible if the exemption code is tied to the same regulation. However, there is precedent for exemption codes being established in this way, so the creation of a 510(k) exemption code for DMHT devices does not preclude use of the billing codes used for reimbursement.

Regardless of what the future holds for future regulatory paths, the advent of TEMPO as a regulated but flexible sandbox for innovation is a significant improvement over the current state of affairs. Mandating a QMS protects patients by ensuring companies do the foundational work to build, monitor, and optimize products against design requirements, even with limited pre-market burden. Quality systems make it more likely that a company that is commercializing with relatively little regulatory oversight is at a minimum doing the foundational work to build a product that addresses the patient need, continuously assess how it is performing against expectations, and implement corrective actions when problems arise. By establishing a 510(k) exemption track for standard tech, the FDA can successfully liberate low risk, well-understood software from the financial burden of costly and complex clinical trials, and enable reimbursement and scaling options that we see in 510(k) codes. Meanwhile, programs like TEMPO can provide a dynamic and highly interactive path for hard-to-regulate technologies without forcing them into the traditional premarket mold. Compared to the wild west of digital health today, this middle approach offers a highly practical regulatory compromise.

On a final, more practical note, the most important takeaway from this discussion is for developers of mental health AI products: FDA policy is a living, breathing thing that is being formed as we speak, now, by the companies who bring LLM-based devices to FDA’s attention and engage with them on how they will be regulated. FDA is forming their opinion on regulatory oversight requirements now, during presubmission meetings, when evaluating TEMPO applications and through interactions with participating companies. The Agency is doing the work now to determine how the unique considerations of LLMs can be incorporated into quality systems, risk management frameworks, and regulatory pathways. The cases we bring forward today are the use cases that will ultimately shape policies and subsequent guidance.

This is precisely why developers can not view policy as something that just happens to them. Pre-submissions, TEMPO participation, Digital Health Advisory Committee meetings, Requests for Information, draft guidance comment periods, and other opportunities for stakeholder engagement all provide mechanisms to influence the regulatory framework that will govern these technologies. Too often, companies remain on the sidelines until policies are finalized. By that point, many of the foundational decisions have been made. For FDA to build momentum behind regulatory approaches that support both public health and innovation, they must be able to demonstrate that those efforts address real stakeholder needs. It is easier for new approaches to gain traction when developers, clinicians, patients, researchers, and investors actively participate and make those needs known. Companies that engage not only gain visibility into emerging regulatory expectations, they help shape them.

Companies must also recognize that quality and regulatory expectations are increasingly becoming a prerequisite for participation, especially in global markets. Across the UK and EU, digital health developers meet expectations for at least foundational quality system processes. For organizations intending to scale internationally or eventually pursue regulated indications, the question is often not whether QMS capabilities will be needed, but when. Companies operating comfortably within today’s enforcement discretion policies cannot assume that position will be there indefinitely. The regulatory framework for AI-enabled mental health products is still being written, and the companies that participate in shaping it today will be best positioned to succeed under it tomorrow. Importantly, FDA’s evolving approach is being informed by companies engaging with the Agency, not by those who stay within the comfortable compliance-free regulatory avoidance of enforcement discretion – a comfort that, as argued here, may not last forever.

## Data Availability

No datasets were generated or analysed during the current study.
